# Age and Serum AMH and FSH Levels as Predictors of the Number of Oocytes Retrieved from Chromosomal Translocation Carriers after Controlled Ovarian Hyperstimulation: Applicability and Limitations

**DOI:** 10.3390/genes12010018

**Published:** 2020-12-25

**Authors:** Yulia V. Shilenkova, Anna A. Pendina, Irina D. Mekina, Olga A. Efimova, Evgeniia M. Komarova, Elena A. Lesik, Mariia A. Ishchuk, Elena M. Fedorova, Olga G. Chiryaeva, Lubov’ I. Petrova, Vera S. Dudkina, Olga E. Talantova, Alexander M. Gzgzyan, Igor Yu. Kogan

**Affiliations:** 1 D.O. Ott Research Institute of Obstetrics, Gynecology and Reproductology, 199034 St. Petersburg, Russia; shil.giulia@gmail.com (Y.V.S.); pendina@mail.ru (A.A.P.); irendf@mail.ru (I.D.M.); evgmkomarova@gmail.com (E.M.K.); lesike@yandex.ru (E.A.L.); mashamazilina@gmail.com (M.A.I.); chiryaeva@mail.ru (O.G.C.); petrovaluba@mail.ru (L.I.P.); dudkinavs@mail.ru (V.S.D.); olga_talantova@mail.ru (O.E.T.); agzgzyan@mail.ru (A.M.G.); ikogan@mail.ru (I.Y.K.); 2 AVA-Peter Clinic, 191014 St. Petersburg, Russia; fedorova-em@avaclinic.ru

**Keywords:** translocation carriers, oocyte retrieval, IVF, PGT-SR, female age, anti-Müllerian hormone (AMH), follicle-stimulating hormone (FSH)

## Abstract

We studied the impact of age and the serum anti-Müllerian hormone (AMH)/follicle-stimulating hormone (FSH) levels on the number of cumulus–oocyte complexes (COCs) retrieved from female reciprocal and Robertsonian translocation carriers after controlled ovarian hyperstimulation (COH). The number of COCs retrieved after COH was retrospectively analyzed in female translocation carriers and 46,XX partners of male translocation carriers from 100 couples. The median number of COCs varied from nine to 16 and did not differ among subgroups of women categorized by age, presence and type of a translocation. The number of COCs correlated negatively with the woman’s age in both the reciprocal and the Robertsonian translocation carriers, while in 46,XX women no correlation was detected. The number of COCs did not differ between the reciprocal and the Robertsonian translocation carriers aged either <35 or ≥35 years. In translocation carriers, the number of COCs correlated with the serum AMH level only in the younger-age subgroups; the correlation was strong positive in reciprocal and moderate positive in Robertsonian translocation carriers. The 46,XX women aged both <35 and ≥35 years showed similar moderate positive correlations. Across all subgroups, the number of COCs correlated moderately negatively with the serum FSH level only in Robertsonian translocation carriers aged <35 years. Our results suggest that chromosomal translocations per se do not increase the risk of poor oocyte retrieval outcome after COH. In translocation carriers, oocyte retrieval outcome depends to a large extent on their age. The serum AMH level strongly predicts oocyte retrieval outcomes only in young reciprocal translocation carriers, while the serum FSH level has a moderate predictive value in young Robertsonian translocation carriers.

## 1. Introduction

A frequent occurrence in the modern world, reproductive disorders are regarded as one of the primary public health challenges. Considering the significant contribution of genetic factors to these disorders [1], in particular, to infertility and pregnancy loss, genomic analysis on different levels of genome organization—from individual genes to chromosome sets—has become an integral part of reproductive medicine.

A common genetic cause of reproductive disorders in a couple is a balanced chromosomal rearrangement in one of the partners. The most widespread and clinically significant chromosomal rearrangements are translocations: reciprocal, an exchange of material between non-homologous chromosomes, and Robertsonian, a centric fusion of two acrocentric autosomes [2]. Characterized by the absence of either gain or loss of genetic material, balanced chromosomal rearrangements do not result in phenotypic changes in their carriers but may, however, lead to the production of chromosomally unbalanced gametes. When unbalanced oocytes and/or spermatozoa participate in fertilization, the resulting embryos are also chromosomally unbalanced. This, in turn, leads to anomalies or arrest of embryonic development and therefore to unfavorable reproductive outcomes, such as infertility, implantation failure during in vitro fertilization (IVF) cycles, pregnancy loss or chromosomally abnormal offspring [3]. To address fertility issues in couples where one of the partners is a translocation carrier, preimplantation genetic testing for structural rearrangement (PGT-SR) is widely used [4,5]. It is in such couples that the optimal number of oocytes retrieved after controlled ovarian hyperstimulation (COH) is key to IVF/PGT-SR efficiency. The probability of producing normal/balanced oocytes is considerably lower in female translocation carriers than in karyotypically normal women: only 1:8 in reciprocal translocation carriers and 1:16 in Robertsonian translocation carriers [3]. Research into the specifics of ovarian response and factors contributing to this process in female translocation carriers is, therefore, a vital measure for the successful conception and birth of a healthy child.

The aim of the present study was to analyze the number of cumulus–oocyte complexes (COCs) retrieved from female reciprocal and Robertsonian translocation carriers after COH and to verify the possible correlation with the woman’s age and the serum level of anti-Müllerian hormone (AMH) and follicle-stimulating hormone (FSH).

## 2. Materials and Methods 

### 2.1. Patients

The study retrospectively included 137 IVF cycles in 100 couples who were referred to the D.O. Ott Research Institute of Obstetrics, Gynecology and Reproductology (St. Petersburg, Russia) or the AVA-Peter Clinic (St. Petersburg, Russia) for IVF/PGT-SR for balanced translocations in the period from March 2009 to May 2019. The number of COCs retrieved after COH in female translocation carriers was compared to that in karyotypically normal partners of male translocation carriers. 

### 2.2. Karyotype Verification

All of the couples referred for IVF/PGT-SR already had a cytogenetic diagnosis. In all of the cases, the initial indications for karyotyping were infertility or pregnancy loss. Prior to the start of an IVF/PGT-SR cycle, cytogenetic diagnoses in all of the translocation carriers were verified by conventional karyotyping followed by fluorescence in situ hybridization (FISH) if required. All conventional karyotyping and FISH procedures including PHA-stimulation of peripheral blood lymphocytes, preparation of metaphase chromosomes, QFH/AcD staining and hybridization were performed according to standard protocols with modifications repeatedly used in our previous studies [6,7,8,9,10]. The final cytogenetic diagnoses are summarized in Table A1. In 52 out of 100 couples, balanced translocation carriers were women (31 cases of reciprocal translocations and 21 cases of Robertsonian translocations). In the remaining 48 couples, balanced translocation carriers were men (27 cases of reciprocal translocations and 21 cases of Robertsonian translocations).

### 2.3. Controlled Ovarian Hyperstimulation

Before inclusion in the first IVF/PGT-SR protocol, all of the female patients were tested for serum levels of day 2–3 AMH and FSH (Table A1). The COH was performed using the antagonist protocol with recombinant and/or urinary gonadotropins. The starting daily dose of gonadotropins ranged from 150 to 300 IU and was chosen based on AMH level, antral follicle count and baseline FSH level. From the sixth day of stimulation, the FSH dosage was adjusted according to the number of growing follicles and their size. COCs were retrieved by transvaginal ultrasound-guided aspiration 36 h after the ovulation trigger administration. A total of 2020 COCs were retrieved in 137 IVF cycles analyzed in this study.

### 2.4. Statistical Analysis

The statistical analysis was performed using The R Project for Statistical Computing (https://www.r-project.org). The inter-group comparison of the patients’ age was performed using the Kruskal–Wallis test. The median numbers of COCs were compared with Mood’s median test. The comparison of the number of COCs between the female reciprocal and Robertsonian translocation carriers <35 years of age and between their counterparts ≥35 years of age was carried out using the Mann–Whitney U test. The correlations between the number of COCs and the patients’ age and between the number of COCs and the serum AMH/FSH levels were measured using Spearman’s rank correlation coefficient. The α-level was set at 0.05.

### 2.5. Ethical Issues

The study was approved by the Ethics Committee of the D.O. Ott Research Institute of Obstetrics, Gynecology and Reproductology. All the patients gave written informed consent to the use of their medical records in the study. The study was conducted in accordance with the Declaration of Helsinki.

## 3. Results and Discussion

Due to a high probability of unbalanced gametes, carriers of balanced translocations have a high risk of infertility, pregnancy loss and the birth of a child with a chromosomal pathology [3,11]. For that reason, should such patients resort to IVF/PGT-SR, it is critical to obtain a sufficient number of oocytes to select the genetically balanced embryos suitable for uterine transfer. Due to a limited number of relevant studies and certain contradictions in their findings [12,13,14], the question of the impact of translocations on oocyte retrieval remains open. In this study, we checked whether female reciprocal and Robertsonian translocation carriers are at risk of a low number of COCs retrieved after COH and whether the number of COCs correlates with the woman’s age and the serum AMH and FSH levels. 

In medical literature, advanced maternal age has been consistently defined as ≥35 years of age. Women aged over 35 years have a dramatically increased risk of aneuploid embryos resulting in miscarriage, stillbirth and offspring with chromosomal pathology [15,16,17]. The dominant reason for this is an elevated probability of meiotic errors in oogenesis and a production of karyotypically abnormal oocytes as a result, both in 46,XX women and in translocation carriers [18]. Therefore, we used an age of 35 years as a cutoff between the young and old age group.

The female patients from the studied couples were subcategorized into six subgroups by age (<35 and ≥35 years of age) and presence of a chromosomal rearrangement (female carriers of a Robertsonian translocation, female carriers of a reciprocal translocation and karyotypically normal partners of male translocation carriers). The subgroups of patients within the groups aged <35 and ≥35 years were checked for age-matching. No statistically significant difference was detected either among the subgroups of reciprocal and Robertsonian female translocation carriers and 46,XX patients aged <35 years (Kruskal–Wallis rank sum statistic H = 0.66979, *p* = 0.7154; the distribution of H approximated by chi-squared distribution with df = 2) or among the subgroups of their counterparts aged ≥35 years (Kruskal–Wallis rank sum statistic H = 0.82567, *p* = 0.6618; the distribution of H approximated by chi-squared distribution with df = 2).

**Figure 1:**
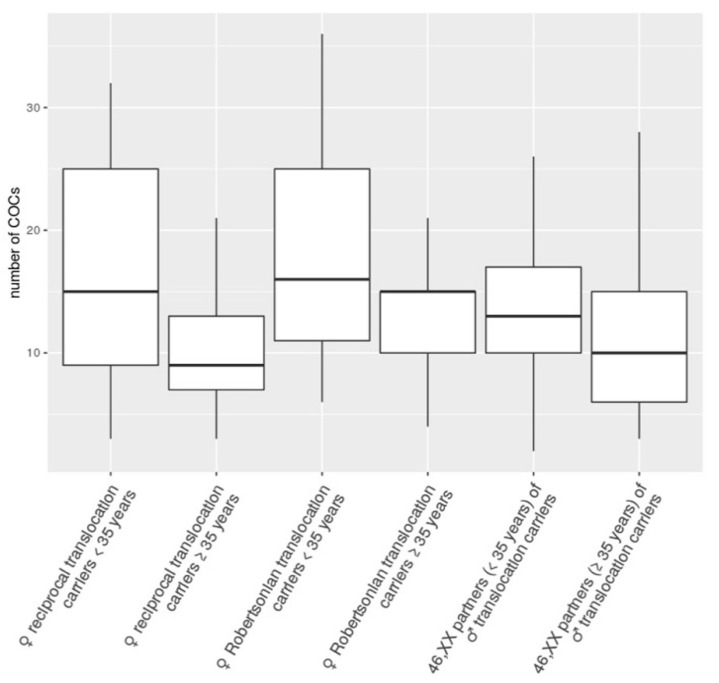
Box plot of the number of cumulus–oocyte complexes (COCs) retrieved after controlled ovarian hyperstimulation in six subgroups of women subcategorized by age (<35 and ≥35 years) and presence of a chromosomal rearrangement (female carriers of a Robertsonian translocation, female carriers of a reciprocal translocation and karyotypically normal partners of male translocation carriers). The lower whisker represents the smallest observation greater than or equal to the lower hinge—1.5 × IQR; the lower hinge represents the 25% quantile; the line through the middle region of the box represents the median, 50% quantile; the upper hinge represents the 75% quantile; the upper whisker represents the largest observation less than or equal to the upper hinge + 1.5 × IQR. The inter-group comparison of the medians showed no statistically significant difference (Mood’s median test, chi-squared = 11.054, df = 7, *p* = 0.1363).

We compared the median numbers of COCs retrieved after COH in IVF/PGT-SR cycles in the above-described six subgroups of women (Figure 1). For each subgroup, we also calculated the interquartile range that reflected the variability of values within the subgroup. The median number of COCs varied from nine to 16 across the subgroups. This number of COCs is categorized as optimal (from 10 to 15), with a suboptimal number varying from four to nine COCs [19]. The highest variability was recorded in the subgroup of female reciprocal translocation carriers aged <35 years (an interquartile range of 16) and the lowest one in the subgroup of Robertsonian translocation carriers aged ≥35 years (an interquartile range of 5). Regardless of a certain decrease in the medians in female translocation carriers ≥35 years of age, the inter-group comparison of the medians showed no statistically significant difference (Mood’s median test, chi-squared = 11.054, df = 7, *p* = 0.1363). Thus, the younger- and the older-age subgroups of both the female translocation carriers and the karyotypically normal partners of male translocation carriers produced an equal median number of COCs that is categorized as optimal. We have therefore concluded that the presence of a balanced translocation per se has no impact on the COH outcome and that the IVF/PGT-SR cycle with own gametes is a procedure of choice for female balanced translocation carriers.

In the literature, the question of ovarian response to COH in female carriers of balanced translocations remains controversial, which is most likely explained by different approaches used by the authors. In the study by Chen et al., who applied up to 450 IU of gonadotropin for stimulation and used serum level of E2 on the day of hCG administration as an indicator of ovarian response, the proportion of very poor responders was significantly higher in the female carriers compared with the partners of male carriers [12]. D’Ippolito et al. reported retrieval of two and one mature oocytes in a female t(1;11) carrier who underwent two ovarian stimulations with high doses of gonadotropins (300 and 400 IU) [20]. The assessment of the ovarian response by follicular output rate (FORT, ratio between the pre-ovulatory follicle count on day of hCG × 100/antral follicle count) revealed poor response in female translocation carriers [14]. The abovementioned results may be associated with alterations of molecular signaling in granulosa cells due to a genetic defect caused by translocation-specific breakpoints [20] and diminished antral follicle sensitivity to FSH [14]. In the studies by Dechanet et al. and Keymolen et al., optimal numbers of COCs were retrieved in female translocation carriers who underwent standard protocols of COH with standard doses of FSH [13,21] which is consistent with our results. However, considering the existing controversy, we have decided to check the possible associations of the oocyte retrieval outcomes in female translocation carriers with the age, type of translocation and the ovarian reserve markers (the serum AMH and FSH levels).

**Figure 2:**
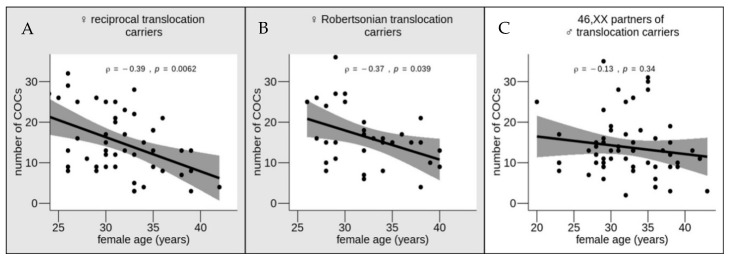
Correlations between the number of cumulus–oocyte complexes (COCs) retrieved after controlled ovarian hyperstimulation and woman’s age in the groups of female reciprocal (**A**) and Robertsonian (**B**) translocation carriers and karyotypically normal partners of male translocation carriers (**C**); Table A1. The statistically significant correlations are shaded in gray (*p* < 0.05, Spearman test).

Using correlation analysis, we checked the strength and the direction of the relationship between the number of COCs and the woman’s age in three groups: female reciprocal translocation carriers, female Robertsonian translocation carriers and karyotypically normal partners of male translocation carriers (Figure 2). In all of the groups, the number of COCs correlated negatively with the woman’s age. However, the correlation reached significance only in the translocation carriers, both reciprocal (ρ = −0.39, *p* = 0.0062) and Robertsonian (ρ = −0.37, *p* = 0.039). In the karyotypically normal women, the correlation was not statistically significant (ρ = −0.13, *p* = 0.34). Thus, it is in female carriers of balanced translocations that age is a significant predictor of the number of oocytes retrieved after COH. It is likely that age-dependent deterioration of the biological control of the physiological and biochemical processes involved in oocyte maturation is enhanced by the presence of chromosomal rearrangement in female translocation carriers. During oogenesis, the germline cells undergo several quality control checkpoints to initiate apoptosis of abnormal cells including those with genetic defects. As translocation carriers have an increased probability to produce chromosomally unbalanced cells, they are at higher risk of drastically depleted overall oocyte production with age than karyotypically normal women.

**Figure 3:**
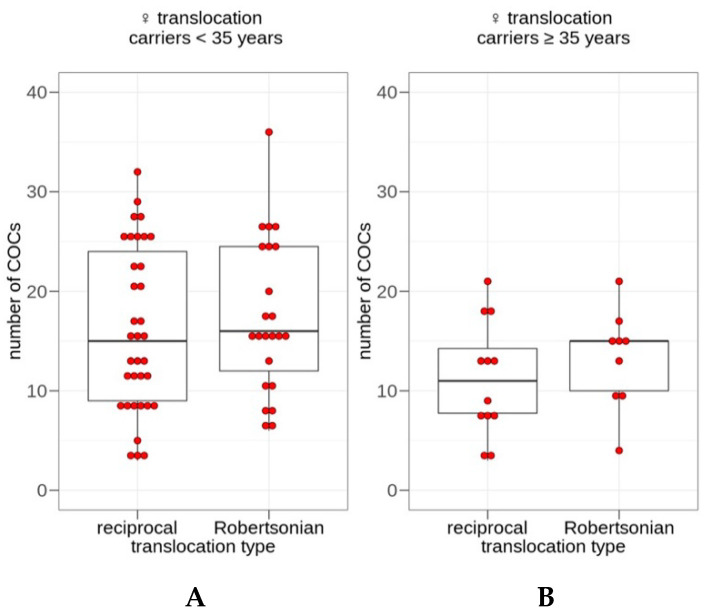
Box plots of the number of cumulus–oocyte complexes (COCs) retrieved after controlled ovarian hyperstimulation in female translocation carriers aged <35 (**A**) and ≥35 (**B**) years. The lower whisker represents the smallest observation greater than or equal to the lower hinge—1.5 × IQR; the lower hinge represents the 25% quantile; the line through the box represents the median, 50% quantile; the upper hinge represents the 75% quantile; the upper whisker represents the largest observation less than or equal to the upper hinge + 1.5 × IQR. No significant difference between the subgroups of female reciprocal and Robertsonian translocation carriers either <35 (**A**) or ≥35 (**B**) years of age was detected (Mann–Whitney U test, U = 364, *p* = 0.5452 and U = 40, *p* = 0.3344, respectively).

At the third stage of the study, we compared the possible effects of reciprocal and Robertsonian translocations on the number of oocytes retrieved from female translocation carriers. Using the Mann–Whitney U test, we compared the number of COCs between the female reciprocal and Robertsonian translocation carriers aged <35 years and between their counterparts aged ≥35 years. No significant difference was detected between reciprocal and Robertsonian translocation carriers aged either <35 years (U = 364, *p* = 0.5452) or ≥35 years (U = 40, *p* = 0.3344; Figure 3). Thus, the translocation type—reciprocal or Robertsonian—does not have a significant impact on the number of oocytes retrieved after COH from female balanced translocation carriers.

Next, we analyzed the possible correlation between the serum AMH and FSH levels and the number of oocytes retrieved after COH from female translocation carriers. The serum AMH and FSH levels are considered to be the ovarian reserve markers. AMH is expressed in granulosa cells of preantral and small antral follicles, i.e., during the growth stage. When the growing follicles reach larger size, AMH expression diminishes and stops once FSH-dependent selection for dominance occurs [22]. Thus, AMH has an important role in regulating the number of growing follicles and may be involved in the determination of the good quality follicles to undergo selection for ovulation or poor quality follicles to be removed through atresia [23]. As the serum AMH and FSH levels in our study were tested in each woman once (before the inclusion in the first IVF/PGT-SR protocol), data from a total of 100 IVF/PGT-SR cycles were initially included in the analysis.

**Figure 4:**
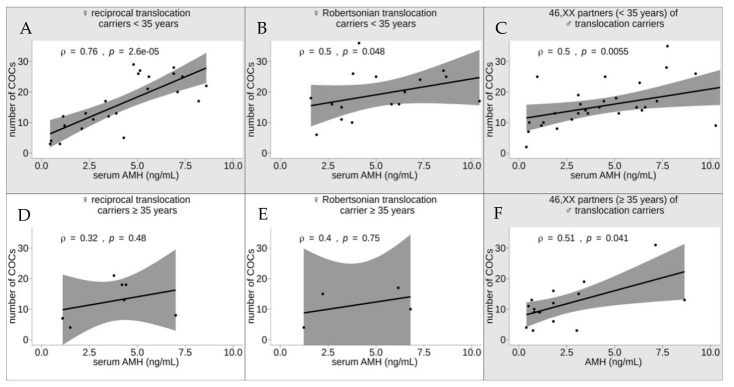
The correlations between the serum anti-Müllerian hormone (AMH) levels and the number of cumulus–oocyte complexes (COCs) retrieved after controlled ovarian hyperstimulation in six subgroups of women subcategorized by age (<35 (**A**–**C**) and ≥35 (**D**–**F**) years) and presence of a chromosomal rearrangement (female carriers of a reciprocal translocation (**A**,**D**), female carriers of a Robertsonian translocation (**B**,**E**) and karyotypically normal partners of male translocation carriers (**C**,**F**)). The statistically significant correlations are shaded in gray (*p* < 0.05, Spearman test).

Using the Spearman test, we performed a correlation analysis of the serum AMH level and the number of COCs in six subgroups of women subcategorized by age (<35 or ≥35 years) and presence of a chromosomal rearrangement (female carriers of a Robertsonian translocation, female carriers of a reciprocal translocation and karyotypically normal partners of male translocation carriers; Figure 4). Six women were excluded from the analysis because their AMH level was equal to or exceeded 15 ng/mL: two female reciprocal translocation carriers aged <35 years, one female Robertsonian translocation carrier aged ≥35 years, one karyotypically normal partner of a male translocation carrier aged <35 years and two karyotypically normal partners (aged ≥35 years) of male translocation carriers. 

The strongest positive correlation between the number of COCs and the serum AMH level was detected in the subgroup of female reciprocal translocation carriers aged <35 years (ρ = 0.76, *p* = 2.6 × 10^−5^). The subgroups of female Robertsonian translocation carriers aged <35 years and karyotypically normal partners (aged <35 years) of male translocation carriers showed correlations of similar strength (ρ = 0.5, *p* = 0.048 and ρ = 0.5, *p* = 0.0055, respectively). No significant correlation was detected in either reciprocal (ρ = 0.32, *p* = 0.38) or Robertsonian (ρ = 0.4, *p* = 0.75) translocation carriers aged ≥35 years. However, to further confirm this finding, a larger group of patients needs to be tested. The subgroup of karyotypically normal women aged ≥35 years, however, showed a rather strong positive correlation (ρ = 0.51, *p* = 0.041; Figure 4). Thus, the serum AMH level strongly predicts the number of COCs retrieved after COH from younger female reciprocal translocation carriers, whereas in female Robertsonian translocation carriers aged <35 years and in karyotypically normal women both <35 and ≥35 years of age, this indicator is less sensitive.

The possible explanation of these results likely comes from a negative selection of unbalanced germline cells, which are produced at high incidence in translocation carriers [3]. Due to apoptosis of a part of unbalanced gametes, the translocation carriers may have a decreased follicle pool compared to karyotypically normal women. However, at the younger age, the follicle pool is not depleted significantly, and high AMH level plays an important role in selection of the dominant follicle. In the older age translocation carriers, the role of AMH is much less pronounced as they have depleted pool of oogenic cells and limited options for choice of the good quality dominant follicles.

**Figure 5:**
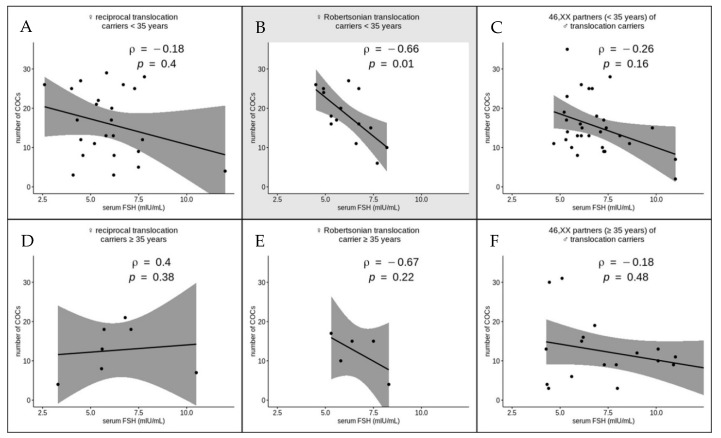
The correlations between the serum follicle-stimulating hormone (FSH) levels and the number of cumulus–oocyte complexes (COCs) retrieved after controlled ovarian hyperstimulation in six subgroups of women subcategorized by age (<35 (**A**–**C**) and ≥35 (**D**–**F**) years) and presence of a chromosomal rearrangement (female carriers of a reciprocal translocation (**A**,**D**), female carriers of a Robertsonian translocation (**B**,**E**) and karyotypically normal partners of male translocation carriers (**C**,**F**)). The statistically significant correlations are shaded in gray (*p* < 0.05, Spearman test).

We performed correlation analysis of the serum FSH level and the number of COCs in the same subgroups of women (Figure 5). Two female Robertsonian translocation carriers were excluded from the analysis because of a significant deviation of their serum FSH level (0.27 and 9.32 mlU/mL). A significant strong negative correlation between the serum FSH level and the number of COCs was detected only in the subgroup of female Robertsonian translocation carriers aged <35 years (ρ = −0.66, *p* = 0.01). Why this correlation is present only in young Robertsonian but not reciprocal translocation carriers is an open and very intriguing question. Further studies will probably shed light on the biological mechanisms underlying this association. In the rest of the analyzed subgroups, the serum FSH level had no impact on the number of COCs retrieved after COH (Figure 5); however, the number of cases with translocation carriers aged ≥35 years was too small to provide meaningful inferences. The increased serum FSH level can, therefore, indicate poor responders to COH only among young female Robertsonian translocation carriers.

Taken together, our results suggest that the prognosis of oocyte retrieval outcome in translocation carriers should first consider female age and then serum AMH and FSH levels.

## 4. Conclusions

Our study demonstrates that the presence of a balanced translocation in woman regardless of the translocation type—reciprocal or Robertsonian—does not decrease the number of COCs retrieved after COH during IVF/PGT-SR cycles. We therefore suggest that the use of IVF followed by PGT-SR in female translocation carriers appears to be practical and justified. It should be taken into consideration, however, that, should a patient undergo an IVF/PGT-SR cycle at an older reproductive age (≥35 years of age), a balanced translocation, either Robertsonian or reciprocal, may still drastically increase the risk of a poor COH outcome. The response to COH in translocation carriers may not differ from that in 46,XX women. However, due to abnormal chromosome conjugation and increased probability of unbalanced gametes, some of which are removed through apoptosis, the depletion of the ovarian pool in translocation carriers progresses faster than in karyotypically normal women. This makes the age especially critical for oocyte retrieval outcome in balanced translocation carriers.

The serum AMH level may be regarded as the strong predictor of a good response to COH only in young (<35 years of age) female reciprocal translocation carriers. The predictive value of the serum AMH level on oocyte retrieval is moderate in young female Robertsonian translocation carriers and karyotypically normal partners of male translocation carriers. In older female translocation carriers, the serum AMH level does not correlate with the number of retrieved oocytes and cannot be regarded as a significant predictor of the COH outcome. It is also necessary to consider that an increased serum FSH level has a negative predictive value of the number of oocytes only in the younger age group of Robertsonian translocation carriers.

The limitations of this study should be mentioned. The first one concerns heterogeneity of the group of reciprocal translocation carriers. Most reciprocal translocations are unique, thus potentially providing a variety of genetic defects caused by translocation breakpoints, which, in turn, may result in very individual effects on ovarian response to COH [20]. The second one is related to a small number of cases with translocation carriers aged ≥35 years. Due to a high risk of reproductive failures, balanced translocation carriers usually have remarkable medical history and are karyotyped at a relatively young age. At genetic counseling, translocation carriers are strongly advised not to delay their reproductive attempts. These issues make the inclusion in the study of a large group of older age translocation carriers highly problematic.

To summarize, either a reciprocal or a Robertsonian chromosomal translocation per se does not increase the risk of a poor COH outcome in an IVF/PGT-SR cycle. The success of the procedure, however, is to a large extent determined by the woman’s age being <35 years, the serum AMH level in younger female reciprocal translocation carriers and the serum FSH level in younger female Robertsonian translocation carriers.

## Data Availability

The raw datasets analyzed during the current study are present in the Table A1 (Appendix A).

## Appendix A

**Table A1:** Clinical characteristics of the patients from the couples referred for in vitro fertilization/preimplantation genetic testing for structural rearrangement (IVF/PGT-SR) for balanced translocations.

Couple #	Carrier’s Karyotype	Partner’s Karyotype	Number of Cycles	Female Age, Years	Number of Cumulus–Oocyte Complexes (COCs)	Serum Anti-Müllerian Hormone (AMH), ng/mL	Serum Follicle-Stimulating Hormone (FSH), mIU/mL
1	46,XX,t(1;16)(p31;q21)	46,XY	2	26	8	2.1	4.6
					9		
2	46,XX,t(2;17)(p21;p13)	46,XY	1	31	9	1.2	7.49
3	46,XX,t(10;15)(q22;q15)	46,XY	3	33	5	4.29	7.49
					12		
				35	9		
4	46,XX,t(10;18)(q21;q22)	46,XY	1	31	12	3.5	4.49
5	46,XX,t(2;11)(p13;q21)	46,XY	2	30	12	1.13	7.7
6	46,XX,t(4;12)(q32;q23)	46,XY	1	33	3	0.96	6.2
7	46,XX,t(2;16)(q33;q22)	46,XY	4	36	8	7	5.57
				38	13		
				39	8		
					3		
8	46,XX,t(3;14)(q25;q11.2)	46,XY	2	29	8	18.75	6.22
					9		
9	46,XX,t(4;11)(q22;q24)	46,XY	1	32	13	3.9	6.18
10	46,XX,t(1;2)(q41;p23)	46,XY	1	28	11	2.7	5.2
	46,XX,t(3;16)(p21.2;p13.3)	46,XY	2	30	17	3.34	6.08
					25		
12	46,XX,t(7;13)(p22;q32)	46,XY	5	26	29	4.8	5.83
					32		
				30	15		
					13		
				32	23		
13	46,XX,t(13;20)(q32;p12)	46,XY	1	31	21	5.54	5.3
14	46,XX,t(5;15)(q15;q25)	46,XY	2	24	27	5.14	4.48
				27	16		
15	46,XX,t(1;22)(q25;13)	46,XY	2	33	22	8.6	5.4
				34	15		
16	46,XX,t(10;13)(q11.2;q14)	46,XY	1	36	21	3.78	6.8
17	46,XX,t(3;8)(q13;p12)	46,XY	1	31	20	7.1	6.1
18	46,XX,t(1;4)(p22;p16)	46,XY	1	35	18	4.2	5.7
19	46,XX,t(11;22)(q23.3;q11.21)	46,XY	1	34	4	0.5	12
20	46,XX,t(5;7)(q35.2;q11.2)	46,XY	1	26	13	2.3	5.8
21	46,XX,t(11;22)(q23;q11.2)	46,XY	1	32	17	8.2	4.3
22	46,XX,t(2;8)(q23;p21)	46,XY	1	35	13	4.31	5.6
23	46,XX,t(1;8)(p22;q21)	46,XY	1	31	25	7.35	7.29
24	46,XX,t(2;17)(p11.2;q11.2)	46,XY	1	35	18	4.41	7.1
25	46,XX,t(4;13)(p16.3;q22.3)	46,XY	1	27	25	5.6	4.01
26	46,XX,t(1;19)(p13;q13.1)	46,XY	2	38	7	1.1	10.5
				39	13		
27	46,XX,t(9;11)(q13;q13)	46,XY	1	33	3	0.45	4.09
28	46,XX,t(6;8)(q12;q11.23)	46,XY	1	29	26	6.9	2.6
29	46,XX,t(5;14)(p15;q24)	46,XY	1	42	4	1.5	3.3
30	46,XX,t(9;10)(p24;q22)	46,XY	1	25	26	5.05	6.7
31	46,XX,t(1;5)(p34;q13)	46,XY	1	33	28	6.9	7.8
32	46,XY, t(1;2)(q41;q34)	46,XX	1	29	15	6.14	6.15
33	46,XY, t(2;9)(q11.2;q34)	46,XX	1	36	16	1.8	6.2
34	46,XY, t(8;18)(q12;p11.2)	46,XX	1	30	19	3.1	5.21
35	46,XY, t(7;11)(q32;q21)	46,XX	1	37	13	8.64	4.27
36	46,XY,t(10;21)(q11.2;q21)	46,XX	1	30	11	11.4	4.67
37	46,XY,t(16;19)(p11.2;p13.1)	46,XX	1	38	19	3.4	6.8
38	46,XY,t(7;21)(q32;q21)	46,XX	2	39	9	1.1	7.3
					10		-
39	46,XY,t(5;11)(p15;q23)	46,XX	1	33	13	3.6	6.5
40	46,XY,t(16;19)(p11.2;p13.1)	46,XX	1	38	15	3.12	6.12
41	46,XY,t(1;7)(q42;p22)	46,XX	1	36	9	1.05	7.92
42	46,XY,t(3;6)(q26.2;q23)	46,XX	3	32	25	4.5	6.5
				34	18		
				35	28		
43	46,XY,t(11;20)(q21;q13)	46,XX	2	33	26	9.22	6.1
					9		
44	46,XY,t(9;15)(q12;q26)	46,XX	1	28	12	18.3	5.3
45	46,XY,t(7;11)(q22;q23)	46,XX	1	38	3	0.74	7.98
46	46,XY,t(3;8)(p25;q12)	46,XX	1	23	17	7.2	5.33
47	46,XY,t(3;13)(p21.2-p21.3;q32)	46,XX	1	38	12	1.8	9
48	46,XY,t(7;11)(q31.2;p14)	46,XX	2	27	13	5.21	5.9
				29	10		
49	46,XY,t(1;8)(q42.1;q24.2)	46,XX	2	29	9	10.27	7.27
				31	13		
50	46,XY,t(6;11)(p21.3;q13)	46,XX	1	35	31	7.13	5.1
51	46,XY,t(1;4)(q32;p16)	46,XX	1	36	6	1.8	5.6
52	46,XY,t(7;14)(q21.3;q31.2)	46,XX	1	29	35	7.75	5.37
53	46,XY, t(3;6)(q25;p21)	46,XX	1	29	11	2.76	8.61
54	46,XY,t(6;16)(q21;q24)	46,XX	1	43	3	3.02	4.4
55	46,XY,t(11;22)(q23.3;q11.2)	46,XX	1	30	18	5.07	6.9
56	46,XY,t(1;17)(p13;p11.2)	46,XX	1	28	10	1.28	7.2
57	46,XY,t(4;8)(q31.3;p21.3)	46,XX	1	33	15	6.59	7.4
58	46,XY,t(1;20)(p36.1;q13.3)	46,XX	1	31	28	7.7	7.6
59	46,XY,t(13;14)(q21.2;p11.2)	46,XX	3	23	10	0.54	5.6
					8		
					10		
60	45,XX,t(13;14)(q10;q10)	46,XY	1	33	16	6.2	0.27
61	45,XX, t(13;14)(q10;q10)	46,XY	1	27	26	3.8	4.5
62	45,XX, t(13;14)(q10;q10)	46,XY	1	32	18	1.6	5.3
63	45,XX,t(13;14)(q10;q10)	46,XY	1	32	17	10.4	5.58
64	45,XX,t(13;14)(q10;q10)	46,XY	2	32	20	6.5	5.8
					13		
65	45,XX,t(13;14)(q10;q10)	46,XY	2	34	16	2.7	5.3
					8		
66	45,XX,t(13;14)(q10;q10)	46,XY	1	35	15	17.5	6.39
67	45,XX,t(14;21)(q10;q10)	46,XY	1	28	24	7.3	4.91
68	45,XX,t(14;21)(q10;q10)	46,XY	2	32	6	1.9	7.7
					7		
69	45,XX,t(14;15)(q10;q10)	46,XY	2	29	11	3.2	6.6
				34	15		
70	45,XX,t(13;14)(q10;q10)	46,XY	3	29	27	8.52	6.21
					15		
				30	27		
71	45,XX,t(13;14)(q10;q10)	46,XY	1	28	15	3.2	7.36
72	45,XX,t(13;14)(q10;q10)	46,XY	1	27	16	5.81	6.74
73	45,XX,t(13;14)(q10;q10)	46,XY	1	26	25	5	4.9
74	45,XX,t(13;14)(q10;q10)	46,XY	1	29	36	4.11	9.32
75	45,XX,t(14;22)(q10;q10)	46,XY	2	28	10	3.75	8.2
		46,XY			8		
76	45,XX,t(13;14)(q10;q10)	46,XY	3	38	15	2.22	7.5
				40	13		
					9		
77	45,XX,t(13;14)(q10;q10)	46,XY	3	36	17	6.16	5.3
				37	15		
				38	21		
78	45,XX,t(13;14)(q10;q10)	46,XY	1	38	4	1.23	8.3
79	45,XX,t(14;21)(q10;q10)	46,XY	1	39	10	6.8	5.8
80	45,XX,t(13;14)(q10;q10)	46,XY	1	30	25	8.66	6.74
81	45,XY,t(14;21)(q10;q10)	46,XX	1	32	17	4.43	7.27
82	45,XY,t(13,14)(q10;q10)	46,XX	1	28	14	6.42	5.38
83	45,XY,t(13;14)(p11.2;p11.2)	46,XX	2	29	9	1.17	7.33
					6		
84	45,XY,t(13;14)(q10;q10)	46,XX	1	33	13	3.1	6.1
85	45,XY,t(14;15)(q10;q10)	46,XX	1	29	16	3.21	6.05
86	45,XY,t(13;14)(q10;q10)	46,XX	1	28	15	4.2	9.8
87	45,XY,t(14;22)(q10;q10)	46,XX	2	31	14	3.48	7.1
				32	11		
88	45,XY,t(14;21)(q10;q10)	46,XX	1	30	13	1.88	8.11
89	45,XY,t(13;14)(q10;q10)	46,XX	1	29	23	6.3	5.36
90	45,XY,t(14;21)(q10;q10)	46,XX	1	38	9	0.8	10.9
91	45,XY,t(14;21)(q10;q10)	46,XX	1	35	10	0.8	10.1
92	45,XY,(15;21)(q10;q10)	46,XX	1	41	13	0.67	10.1
93	45,XY,t(13;14)(q10;q10)	46,XX	1	42	11	0.5	11
94	45,XY,t(13;14)(q10;q10)	46,XX	1	20	25	0.96	6.67
95	45,XY,t(13;14)(q10;q10)	46,XX	1	36	4	0.39	11.5
96	45,XY,t(13;14)(q10;q10)	46,XX	1	33	8	1.99	5.9
97	45,XY,t(13;14)(q10;q10)	46,XX	1	36	4	17.33	4.32
98	45,XY,t(13;14)(q10;q10)	46,XX	1	27	7	0.5	11
99	45,XY(13;14)(q10;q10)	46,XX	1	32	2	0.39	11
100	45,XY(13;14)(q10;q10)	46,XX	1	35	30	15	4.44
